# Optimization of total phenolic content from *Terminalia chebula* Retz. fruits using response surface methodology and evaluation of their antioxidant activities

**DOI:** 10.1371/journal.pone.0202368

**Published:** 2018-08-14

**Authors:** Zunlai Sheng, Jiahong Zhao, Ishfaq Muhammad, Ying Zhang

**Affiliations:** College of Veterinary Medicine, Northeast Agricultural University, Harbin, China; College of Agricultural Sciences, UNITED STATES

## Abstract

Ultrasonic-assisted extraction (UAE), using aqueous ethanol as the solvent, was firstly applied to extract phenolic compounds from *Terminalia chebula* Retz. fruits (*T*. *chebula* fruits). In this study, ethanol concentration (%), ultrasonic intensity (W/cm^2^), particle diameter (mm), extraction temperature (°C), ultrasonic time (min), liquid-solid ratio (mL/g) and extraction cycle were investigated by single-factor experiment and then optimized using a Box-Behnken design. The optimized result for UAE was 68% ethanol concentration, ultrasonic intensity of 3.6 W/cm^2^, solid-liquid ratio of 23 mg/mL, particle size of 0.18 mm and ultrasonic time of 20 min for 2 times at 70 °C. The yield of total phenolic was 448.7 ± 2.15 mg GAE/g DW under the above optimum conditions, which agreed with the predicted value (447.8 mg GAE/g DW). Compared to conventional solvent extraction (CSE), UAE extracts showed excellent DPPH radical, DPPH, ABTS scavenging activities and reducing power in a dose-dependent manner, and better than that of CSE extracts. Additionally, the extract of the *T*. *chebula* fruits was analyzed by HPLC-ESI/MS. In summary, UAE could effectively extract phenolic compounds from *T*. *chebula* fruits. In addition, the extract could be used as a potential source of natural antioxidants.

## Introduction

*Terminalia chebula* Retz. (*T*. *chebula*) found in the tropical areas of the world and commonly called *Chebulae Fructus* (Hezi) in China. The plant belongs to the family Combretaceae [1]. It have traditionally been utilized as medicines for relieving bleeding piles, diarrhea, hiccoughing, sore throat and bladder diseases [2]. It has been reported that *T*. *chebula* fruits rich in phenolic compounds such as gallic acid (GA), ellagic acid (EA) and corilagin (CG). These compounds are powerful antioxidant, anti-inflammatory, cardiotonic, antibacterial and anticarcinogenic [3]. Based on the pharmacological activities of *T*. *chebula* fruits, it can be used as an important source to extract natural phenolic compounds. Hence, it is essential to optimize and develop a reliable extraction method to obtain high yield of phenolic compounds from *T*. *chebula* fruits.

Currently, natural antioxidants from herbs are attracting increasing attention for their potential utility. The active phenols and antioxidants from *T*. *chebula* fruits may be obtained by an extraction process for potential use in functional foods or nutraceuticals. Several extraction techniques have been employed for the extraction of phenolic compounds from *T*. *Chebula* fruits, such as reflux system in combination with water-ethanol and water-propylene glycol [4], and subcritical water extraction [5]. However, some antioxidant activities are usually decreased using traditionally extraction methods due to high temperature and long treatment time. Chemat et at. [6] have pointed the benefits of using ultrasonic treatment in food processing to decrease process energy, save time and increase shelf life. Ultrasonic-assisted extraction (UAE) has been reported to enhance dissolution of effective components by disrupting cell tissue, such as extracting Lutein and β-Carotene from Spinach [7] and total phenol from Spinach [8]. It has been reported that thermal function of ultrasound has effects on plant cells and tissues due to the fact that ultrasonic waves could produce heat and absorbed by herbs tissues [9, 10]. Additionally, ultrasound has mechanical effect via acoustic assisted cavitation along with efficient mass transfer of the cell content to the solvent due to collapse of bubbles [7, 11].

UAE has not been reported for extraction of total phenolics (TP) from *T*. *chebula* fruits, but may enhance yield or antioxidant activities over traditional solvent extraction methods. The objectives of this study were to (1) optimize the UAE process of phenolic compounds from *T*. *chebula* fruits by response surface methodology; (2) compare extraction yields and antioxidant activities from UAE and a traditional solvent extraction method; and (3) do phytochemical analyses of extract of *T*. *chebula* fruits using HPLC-ESI/MS.

## Materials and methods

### Materials

The fruits of *T*. *chebula* were bought from Shiyitang herbal Pieces limited liability Company (Harbin, China) in 2017. It was identified and authenticated by Associate Professor Junkai Wu at Heilongjiang University of Chinese Medicine, Harbin, Heilongjiang Province, China. The fruits which had reached physical maturity (yellowish green) were selected. The dried fruits were ground and sieved by the prescription sieves which was standardized by Pharmacopoeia of People’s Republic of China (2015). The lisenced specimens (Accession no. 1009011ch) have been well placed in the Herbarium of College of Veterinary Medicine (Northeast Agricultural University).

Reference substances (Gallic acid and Ascorbic acid; purity > 98% (w/w)) were both acquired from the Chinese Institute for the Control of Pharmaceutical and Biological Products (Beijing, China). Folin-Phenol, 2,4,6-Tris (2-pyridyl)-s-triazine (TPTZ), 1,1-Diphenyl-2-picrylhydrazyl (DPPH), Trichloro acetic acid (TCA), [2,2'-azino-bis-(3-ethylbenzthiazoline-6-sulfonic acid)] ABTS, Ferric chloride, Riboflavin, 6-Hydroxy-2,5,7,8-tetramethylchroman-2-carboxylic Acid (Trolox), Nitro blue tetrazolium (NBT), and Methionine were obtained from Sigma Chemical Co. (St. Louis, USA). The experiments were performed with purified distilled water obtained from Milli-Q academic water purification system (Millipore, Bedford, MA, USA). All other reagents were of analytical grade.

### Quantification of total phenolic content

The quantification of total phenols in *T*. *chebula* fruit extracts were measured by Folin-Ciocalteu method and gallic acid was used as a reference substance [12]. Briefly, 0.1 mL of the gallic acid standard solution (0.05, 0.08, 0.10, 0.14, 0.17 and 0.2 mg/mL) or sample solution intermingled with distilled water (50 mL), Folin–Ciocalteu reagent (5 mL) and 1.5 mL of Na_2_CO_3_, and mixed in a brown volumetric flask. The reaction mixture was measured at 765 nm after incubation in the dark for 3 h. Linear regression method was applied for quantification through a six-point calibration curve. The TP content was then expressed as mg of Gallic acid equivalents per g of dry weight (DW).

### Ultrasound-assisted extraction (UAE) optimization

**Fig 1:**
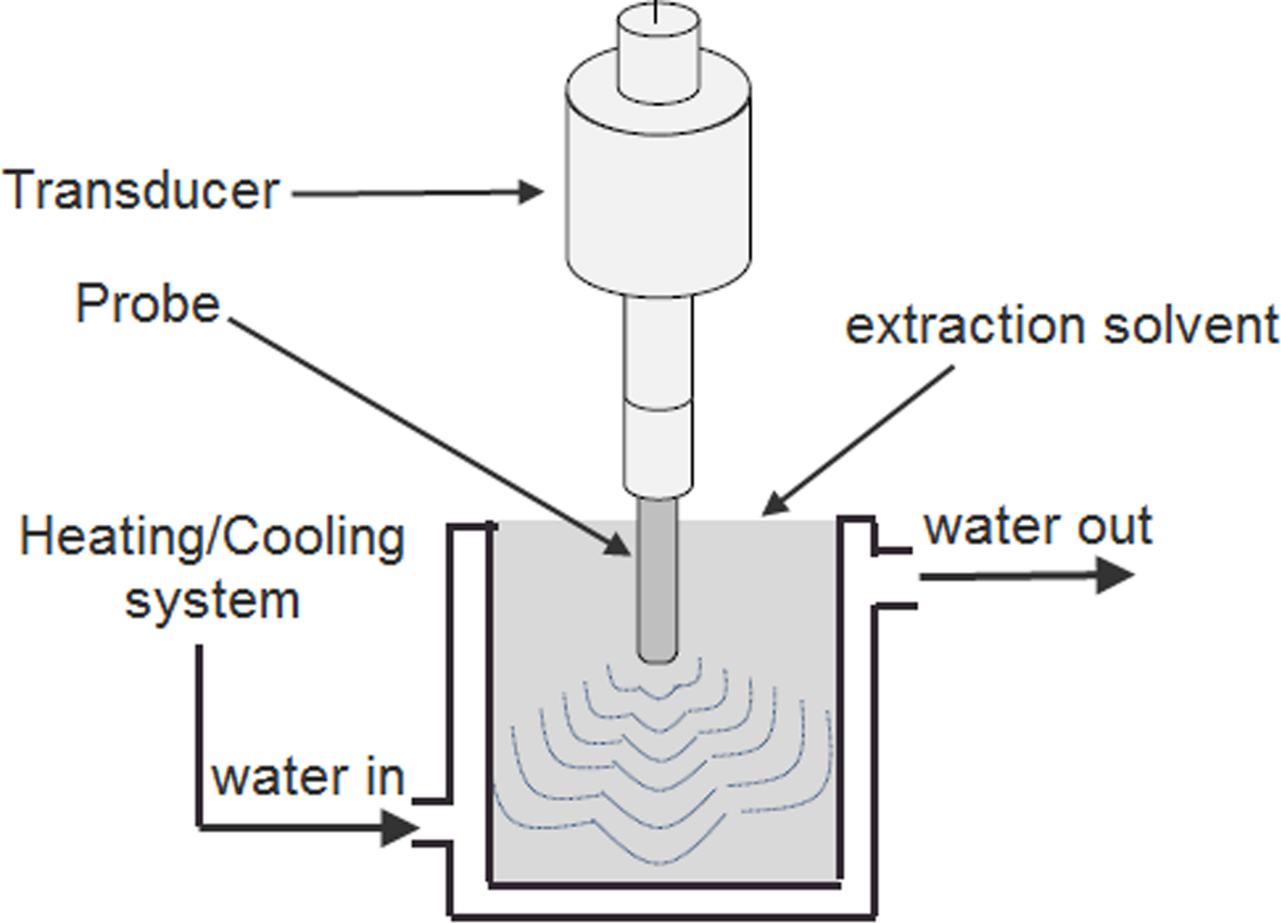
Diagram of the UAE system.

BILON-S650CT high-power ultrasonic processor (Shanghai Binlon Instrument CO., LTD, China) employed for ultrasonic extraction in a toughened glass tank in combination with an ultrasonic transducer (30 kHz, 650 W) and a power meter for changing the parameters during ultrasonic operation. The extraction temperature was maintained using a double layered extraction cells by cooling/heating systems (Fig 1).

A 12 mm acoustic horn (frequency range: 25–30 kHz; power range: 60–650 W; crushing capacity: 50–150 mL) was used to treat the solution with ultrasound placed 1 cm from the top of the extraction cell. The on/off time for the ultrasonic device was noted to be 1 s and 2 s, respectively. Taking into account the dissipated heat, ultrasonic power was calculated using calorimetric measurement and was expressed as ultrasonic intensity (UI) by using Eq (1) [13, 14]:

$$UI=\frac{4P}{\pi D^{2}}$$ (1)

Where UI represents ultrasonic intensity (W/cm^2^), D represents ultrasonic reactor internal diameter (cm), and P is the value of power which is calculated according to the Eq (2) [13, 14].

$$P=mC_{p}\frac{dT}{dt}$$ (2)

Where P is the input power, m represents mass of the solvent (g), C_p_ (heat capacity) of the solvent in kJ/kg °C, and dT/dt is the temperature variation according to time (°C/s).

The value of input powers were adjusted to 100, 200, 300, 400, 500, and 600 W in single-factor experiments which were equivalent to 1.6, 3.3, 5.0, 6.7, 8.5 and 10.0 W/cm^2^, respectively. Single-factor experiments were performed at different conditions: ethanol concentration (0, 20, 40, 60, 80 and 95% (v/v)), ultrasonic intensity (0, 1.6, 3.3, 5.0, 6.7, 8.5 and 10.0 W/cm^2^), the range of particle diameters (0.15–2.00 mm) were determined by the prescription sieves (No. 1–9) which were standardized by Pharmacopoeia of People’s Republic of China (2015), 30, 40, 50, 60, 70 and 80 °C were taken as extraction temperatures, ultrasonic time (10, 15, 20, 25 and 35 min), liquid-solid ratio (10, 15, 20, 25, 30 and 35 mL/g) and the number of extraction chosen from 1 to 6. These experiments were repeated thrice. Finally, dried extracts were obtained following vacuum drying.

According to single-factor test, the parameters which affected the extraction process of TP from *T*. *chebula* fruits were optimized using a 17-run Box-Behnken Design (BBD). In addition, 12 factorial points and 5 axial points were used to determine the optimal extracting conditions. Three variables (X_1_, concentration of ethanol; X_2_, Ultrasonic intensity; X_3_, solid-liquid ratio) and three levels were given the values as 1 (high), 0 (intermediate) and −1 (low), are shown in Table 1. The formula of second-order polynomial mode is given below:

$$Y=\beta_{0}+\sum_{i=1}^{3}\beta_{i}X_{i}+\sum_{i=1}^{3}\beta_{ii}X_{i}^{2}+\sum_{i=1}^{2}\sum_{j=i+1}^{3}\beta_{ij}X_{i}X_{j}$$ (3)

whereas Y represents the response; β_0_ was a constant; β_i (_linear_)_, β_ii_ (quadratic) and β_ij_ (interactive) were the coefficients; *X*_i_ denotes independent variables. The terms *X*_i_*X*_j_ and *X*_i_^2^ represented the quadratic and interaction terms, respectively.

**Table 1:** Variables and experimental design levels for RSM.

Independent variables	Coded symbols	Levels		
		-1	0	1
Ethanol concentration (%)	X_1_	40	60	80
Ultrasonic intensity (W/cm^2^)	X_2_	1.6	3.3	5.0
Solid-liquid ratio (mg/mL)	X_3_	20	25	30

### Conventional reflux extraction (CRE)

The reflux extraction of TP from the dried ripe *T*. *chebula* fruits was assessed by a previously described method [4]. The extraction parameters were temperature of 76 °C, ethanol concentration of 76.4%, liquid to solid ratio of 150 (mL/g), and time duration of 82 min. Additionally, extraction cycle 2 was used to exhaust the TP from the material. The extract was filtered and dried under vacuum.

### In vitro antioxidant activity

#### Reducing power assay

The reducing power was assessed by a previously described method with some modifications [15]. The reaction containing 1.0 mL of UAE extract solution or CRE extract solution (0.03–0.08 mg/mL), 2.5 ml of K_3_Fe(CN)_6_ (1%, w/v) and 2.5 mL of 0.2 M Na_2_HPO_4_ (sodium phosphate buffer, pH 6.6) at 50 °C for 20 min. Then, Trichloroacetic acid (2.5 mL, 10%, w/v) was incorporated into the mixture and centrifuged at 5000 rpm for 10 min. Finally, 0.5 mL of Ferric chloride (0.1%) and the upper layer (5 mL) were mixed together. The absorbance was measured at 700 nm against a blank control (reaction mixture in which the solvent is used instead of sample solution) after 10 min. While, ascorbic acid was taken as positive control.

#### Ferric reducing antioxidant power (FRAP)

The reaction mixture containing 500 μL of the UAE extract solution or CRE extract solution (0.01–0.025 mg/mL), and FRAP reagent (5 mL) was incubated at 37 °C for 30 min. After incubation period, absorbance was measured against a blank control at 700 nm. Ascorbic acid was used as the positive control. FRAP reagent was made up of 0.3 M pH 3.6 Sodium acetate buffer, 10 mM TPTZ solution, 20 mM Ferric chloride solution in a volume ratio of 10:1:1. FRAP solution was standardized against FeSO4·7H_2_O (0.10, 0.14, 0.18, 0.22, 0.26 and 0.30 mM) and expressed as mM FeSO4·7H_2_O per g material.

#### DPPH radical scavenging assay

A previous method was used to measure DPPH radical scavenging activity as mentioned in literature [16] with some modifications. The reaction mixture containing 2.5 mL UAE extract solution or CRE extract solution (0.001–0.011 mg/mL) and 3.5 mL of 0.1 mM DPPH, kept in the dark at room temperature for 1 h. The absorbance of the sample and positive control (ascorbic acid) was measured against a blank control at 517 nm. The following formula was used to calculate DPPH radical scavenging activity.

$$\mathrm{Scavenging}\;\mathrm{activity}\;(\%)=\frac{A_{s}-A_{i}}{A_{s}}\times 100$$ (4)

where As represents the absorbance of distilled water alone, Ai is the absorbance of sample of different concentrations. The IC_50_ value shows that the concentration of the extract inhibited 50% DPPH radical formation.

#### ABTS radical scavenging assay

ABTS radical scavenging ability of the sample at different concentrations was determined as described previously with minor modifications [17]. 7 mM ABTS and 2.45 mM potassium persulfate reacted together in the dark at room temperature for 16 h to produce ABTS radical cation (ABTS+). 80% ethanol was mixed with ABTS^+^ solution to dilute it in order to obtain an absorbance of 0.700 ± 0.005 at 734 nm. The reaction mixture including 0.5 mL UAE extract solution or CRE extract solution (0.07–0.17 mg/mL) and ABTS^+^ solution (2 mL) was incubated at 37 °C for 30 min and examined against a blank control at 734 nm along with a positive control (ascorbic acid). The average value was calculated from three experimental replicates. The formula used for ABTS radical scavenging activity is as follows:

$$\mathrm{Scavenging}\;\mathrm{activity}\;\left(\%\right)=\frac{A_{s}-A_{i}}{A_{s}}\times 100$$ (5)

where A_s_ represents the absorbance of distilled water alone, A_i_ is the absorbance of sample of different concentrations. The IC_50_ value shows 50% inhibition of ABTS radical formation.

#### Superoxide radical scavenging assay

A previous method was applied to measure superoxide radical scavenging activity assay of TCFE with minor modifications [18]. Briefly, the mixture including 50 mM Tris–HCl buffer (4.5 mL, pH 8.2), 2 mL distilled water, 2 ml of UAE extract solution or CRE extract solution (0.01–0.05 mg/mL) and 0.5 mL of 25 mM pyrogallol solution was placed in an incubator at 37 °C for 5 min. Finally, 1.0 mL HCl (10 mM) was added to terminate the reaction. The absorbance of the reaction mixture and ascorbic acid (positive control) was noted against a blank control at 560 nm. The scavenging percentage was determined based on the formula:

$$\mathrm{Scavenging}\;\mathrm{activity}\;\left(\%\right)=\left[1-\frac{A_{1}-A_{2}}{A_{0}}\right]\times 100$$ (6)

where A_0_ denotes the absorbance of the control (distilled water), A_1_ represents absorbance of the sample, and A_2_ is the absorbance of the sample only (Tris–HCl buffer instead of pyrogallol solution). The IC_50_ value shows 50% inhibition of superoxide radical scavenging formation.

### Identification of phenolic compounds

The HPLC/DAD/ESI-MS analysis was performed on an Agilent 1100 HPLC equipped with a Diode Array Detection (DAD) and an HP 1100 MSD API-electrospray (Agilent Technologies) operating both in positive and negative ionization mode.

Gemini C_18_ column (250 mm × 4.6 mm, 5 μm, Phenomenex analytical instruments Co., Ltd., America) was used for *T*. *chebula* fruit extract analysis. Mobile phase consisted of 0.1% formic acid (A) and methanol (B) at constant flow of 0.8 mL/min. The solvent gradient elution schedule was as follows: 0–12 min, 10–90% B; 12–14 min, 90–90% B; 14–14.1 min, 90–10%; 14.1–16 min, 10–10%. The injection volume was 10 μL and the column temperature was kept at 30 °C. The detection wavelength was kept at 254 nm.

Mass spectrometer operating conditions were: drying gas temperature of 350 °C at a flow rate of 10 L/min; nebulizer pressure of 30 psi; sheath gas temperature of 250 °C at a flow rate of 7 L/min; fragmenter voltage of 100 V; capillary voltage of 2500 V; and mass range of 100–1500 D.

### Statistical analysis

All the experiments were repeated three times and values were expressed as means ± SD (n = 3). Stat-Ease Design-Expert 7.0.0 (Trial version, Stat-Ease Inc., Minneanopolis, MN, USA) software was employed for the experimental design and statistical analysis of response surface methodology. Mean values were regarded as significantly different at *p* < 0.05. Statistical analysis of the experimental data of the antioxidant tests were carried out using ANOVA (one-way analysis of variance) followed by Tukey test. SPSS software (version 18.0, SPSS Inc., Chicago, IL, USA) was used for the analysis data.

## Results and discussion

### Single-factor experimental analyses

#### Influence of ethanol concentration on TP extraction

The concentration of solvent is an important factor in solid-liquid extraction system. Importantly, properties such as dielectric constant, surface tension, polarity, consistency, vapor pressure and solvent penetration and interaction with plant matrix have important effects on cavitation effect involved in UAE process [19]. Notably, ethanol concentration was set at 0, 20%, 40%, 60%, 80% and 100% to test the influence of different ethanol concentration on TP extraction. Other reaction conditions were as follows: ultrasonic intensity 5 W/cm^2^, particle diameters 0.25 mm, extraction temperature 60 °C, ultrasonic time 20 min, liquid-solid ratio 20 mL/g and extraction cycle 2.

**Fig 2:**
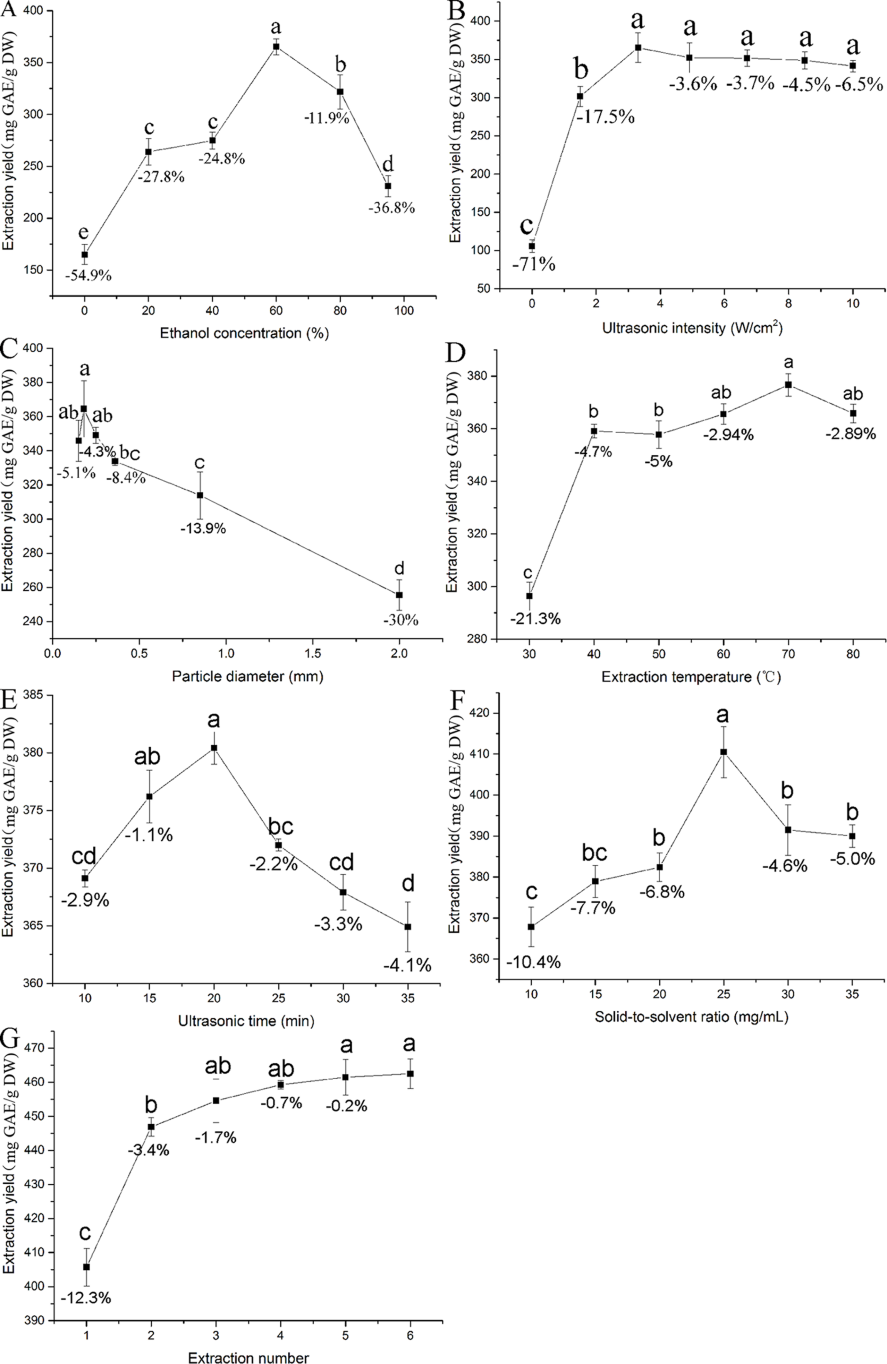
Effects of different extraction parameters (ethanol concentration, %; ultrasonic intensity, W/cm^2^; particle diameter, mm; extraction temperature, °C; ultrasonic time, min; solid-liquid ratio, mg/mL and extraction cycle) on yield of total phenolics from the *T*. *chebula* fruits. Values were expressed as mean ± SD (n = 3), and evaluated by one-way AVONA followed by the Tukey test. Different letter and same letter were considered to be statistically significant (*p* < 0.05) and statistically insignificant (*p* > 0.05), respectively.

Fig 2A showed that 60% ethanol gives the highest extraction yield, while water gives the lowest extraction yield in UAE process. Other reports have also found that 60% ethanol was suitable to extract TP or other phenolic compounds from *T*. *chebula* fruits using the reflux method [20]. Similar extraction yield of TP from *T*. *chebula* fruits was obtained using the same ethanol concentration by the reflux method and UAE, respectively. A remarkable extraction yield difference (*p* < 0.05) was found among the solvents with 60% ethanol concentration versus other concentrations, with the former giving 11.9–24.8% higher yield than 40% and 80% ethanol, respectively (Fig 2A). Therefore, an ethanol concentration of 60% was chosen for subsequent RSM experiments.

#### Influence of ultrasonic intensity on TP extraction

Ultrasonic power is defined as ultrasonic intensity, which involves the cavitation effect. We tested seven levels of ultrasonic intensity ranged from 0–10 W/cm^2^ to examine the effects on TP extraction yield. Other parameters are set as follows: ethanol concentration 60%, particle diameters 0.25 mm, extraction temperature 60 °C, ultrasonic time 20 min, liquid-solid ratio 20 mL/g and extraction cycle 2.

Fig 2B showed that increase in ultrasonic intensity from 0 to 3.3 W/cm^2^ enhances the yield of TP. The results revealed that increase in ultrasonic intensity enhances the cavitation effect which in turn facilitated the disruption of cell walls [21]. However, the extraction yield of TP declined when the ultrasonic intensity was over 3.3 W/cm^2^. The decrease in extraction yield with ultrasonic intensity may be due to the degradation of TP and cavitation effect. Intriguingly, TP were unstable and would be degraded under high ultrasonic intensity [22]. Significant (*p* < 0.05) differences in extraction yield were found among the ultrasonic intensities with the value of 3.3 W/cm^2^ versus the value of 1.6 W/cm^2^, the former giving 3.6–17.5% higher yield than the both experimental neighbors (Fig 2B). Hence, the ultrasonic intensity of 3.3 W/cm^2^ was selected for subsequent RSM experiments.

#### Effect of particle size on TP extraction

It is well known that particle size is another important parameter in solid-liquid extraction system. Generally, it has been previously reported that reduction in particle size leads to increase in the extraction yield due to increase in the surface area available for contact with the solvent [23]. In this study, *T*. *chebula* fruits sizes (varied from 0.15 to 2 mm) were used to investigate the influence of particle size on extraction yield. Other parameters are set as follows: ethanol concentration 60%, ultrasonic intensity 3.3 W/cm^2^, extraction temperature 60 °C, ultrasonic time 20 min, liquid-solid ratio 20 mL/g and extraction cycle 2.

Fig 2C showed that decreasing mean particle size causes an increase in the extraction yield of TP until the mean particle size reached 0.18 mm, which proved that a decrease in the particle size was beneficial to the migration of components from solid to the liquid. However, it was reported previously that smaller particle size leads to difficulty in extraction due to laborious diffusion [24]. Additionally, Fig 2C displayed that the yield of TP was slightly reduced when the particle size decreased from 0.18 to 0.15 mm. Similarly, it has been reported in other studies while extracting active compounds from herbs [25]. Compared to 0.18 mm particle size, the extraction yield of TP from *T*. *chebula* fruits decreased by 4.3 and 5.1% at the particle size of 0.15 and 0.25 mm respectively, but there was no marked statistical difference (*p* > 0.05) among the particle sizes with the value of 0.18 mm versus the value of both experimental neighbors. Thus, particle size of 0.18 mm was selected for the following experiments.

#### Influence of temperature on TP extraction

The extraction yield of TP was tested at extraction temperature from 30 to 80 °C. Other parameters are set as follows: ethanol concentration 60%, ultrasonic intensity 3.3 W/cm^2^, particle size 0.18 mm, ultrasonic time 20 min, liquid-solid ratio 20 mL/g and extraction cycle 2. It has been noted that increasing temperature causes an increase in the extraction yield of TP. The peak yield (376.3 mg GAE/g DW) was obtained at an extraction temperature of 70 °C. However, the extraction yield slightly decreased with further increase in extraction temperature (Fig 2D). It has been speculated that the combination of thermal and cavitation effects play a crucial role in UAE. The rise in temperature had a positive influence on extraction yield due to the reason that it accelerated the molecular movement and decreased the solvent viscidity [26, 27].

However, it should be kept in mind that high temperature could cause the degradation of the phenolic compounds [28]. Therefore, the increase in temperature could have both positive and negative effects. This finding was agreed with Altemimi who found that the thermal degradation of flavonoids and the decrease of number of acoustic cavitation bubbles were lead to decrease the amount of quercetin and rutin [29]. Importantly, no statistical significance (*p* > 0.05) was found among the extraction temperatures with the value of 70 °C versus the value of both experimental neighbors, thus a temperature of 70 °C was selected for the following experiments.

#### Effect of ultrasonic time on TP extraction

The extraction yield of TP was tested at ultrasonic time from 10 to 35 min. Other parameters are set as follows: ethanol concentration 60%, ultrasonic intensity 3.3 W/cm^2^, particle size 0.18 mm, extraction temperature 70 °C, liquid-solid ratio 20 mL/g and extraction cycle 2. Fig 2E represents the effect of ultrasonic time on the extraction yield of TP. The extraction yield increases from 10 to 20 min and reduced from 20 to 35 min. It was due to the fact that the release of bioactive compounds from the matrix cell walls could be accelerated. So the extraction yield of TP was increased by 20 min. However, extraction yield decreases and degradation of TP increases when the time interval exceeds 20 min. Surprisingly, the difference between the peak yield (380.1 mg GAE/g DW) and the adjacent yield (372.4 mg GAE/g DW) were statistically significant (*P* < 0.05), but minimum changes (2.2%) were observed in the yield of TP. Thus, an ultrasonic time of 20 min was chosen for subsequent tests.

#### Effect of solid-liquid ratio on TP extraction

The extraction yield of TP was tested at solid-liquid ratio from 10 to 35 mL/g. Other parameters are set as follows: ethanol concentration 60%, ultrasonic intensity 3.3 W/cm^2^, particle size 0.18 mm, extraction temperature 70 °C, liquid-solid ratio 20 mL/g and extraction cycle 2.

Fig 2F represents the yields of TP remained constant up to 25 mL/g, but decreases with increase in volume. Similarly, Sun *et al*. demonstrated that diffusion of the solvent into cells enhanced with the rise of solid-liquid ratio [24]. However, too high liquid-solid ratio can restrain the generation of aerosol and the cavitation effect. Similar results have been reported by Lu et al., who demonstrated that an increase in solvent volume did not boost yield [30]. Fig 2F also showed statistical significance (*p* < 0.05) among the liquid-solid ratio with the value of 25 mg/mL versus both experimental neighbors, the former giving 4.6–6.8% higher yield. Hence, 25 mg/mL was adopted as the center point for subsequent RSM experiments.

#### Effect of extraction cycle on TP extraction

Fig 2G showed that doubling the extraction cycle from 1 to 2 enhances significantly (*p* < 0.05) the yield of TP. The yield of TP extraction from extraction cycle 2 to 6 has slowly paced down. It has been found that there was no statistical difference (*p* > 0.05) between the extraction cycles with the value of 2 versus the value of 3, which indicated that TP has been completely extracted from the plant. In view of the extraction efficiency and energy cost, the extraction cycle 2 was chosen as the suitable extraction cycle.

### Optimization of extraction conditions using RSM

#### Fitting the model

Box-Behnken design (BBD) was used to optimize the three parameters for optimum extraction conditions. The response values and predicted values were obtained from 17 runs and factorial design, respectively (Table 2). The minimal difference (0–0.44%) between the predicted values and actual values proved the accuracy of the model. Multiple regressions applied for the analysis of the data and relativity between the test variable and response variable which was described by second-order polynomial equation:

$$Y=444.26+9.2X_{1}+8.18X_{2}-6.27X_{3}+4.17X_{1}X_{2}-4.58X_{1}X_{3}-1.78X_{2}X_{3}-15.02X_{1}^{2}-25.52X_{2}^{2}-13.37X_{3}^{2}$$ (7)

where *X*_*1*_, *X*_*2*_ and *X*_*3*_ were represents ethanol concentration, ultrasonic intensity and solid-liquid ratio, respectively.

**Table 2:** The Box-Behnken experimental design with four independent variables.

No.	X_1_ (%)	X_2_ (W/cm^2^)	X_3_ (mg/mL)	Actual value (mg GAE/g DW)	Predicted value (mg GAE/g DW)	Difference (%)
1	40	1.6	25	388.8	390.52	-0.44
2	80	1.6	25	401.1	400.58	0.13
3	40	5.0	25	398	398.53	-0.13
4	80	5.0	25	427	425.27	0.41
5	40	3.3	20	408.9	408.38	0.13
6	80	3.3	20	434.2	435.93	-0.40
7	40	3.3	30	406.7	404.98	0.42
8	80	3.3	30	413.7	414.23	-0.13
9	60	1.6	20	402.9	401.70	0.30
10	60	5.0	20	421.6	421.60	0
11	60	1.6	30	392.7	392.70	0
12	60	5.0	30	404.3	405.50	-0.30
13	60	3.3	25	444.6	444.26	0.08
14	60	3.3	25	445.3	444.26	0.23
15	60	3.3	25	442.4	444.26	-0.42
16	60	3.3	25	443.8	444.26	-0.10
17	60	3.3	25	445.2	444.26	0.21

ANOVA results (Table 3) showed that the Model *p*-value was remarkably significant (less than 0.0001). Significant model terms were *X*_*1*_, *X*_*2*_, *X*_*3*_, *X*_*1*_*X*_*2*_, *X*_*1*_*X*_*3*_, *X*_*1*_^*2*^, *X*_*2*_^*2*^ and *X*_*3*_^*2*^ and there could be a chance of 0.01% that model “*F*-value” becomes large due to noise. The value of Lack of Fit is 3.68, not significant to the pure error. The chance of error in the "Lack of Fit F-value" was 12.01% due to noise signals. The value of "Adj R-Squared" was 0.9925 which is in agreement with "Pred R-Squared" value (0.9602). The noise ratio 39.85 (calculated by "Adeq Precision") showed an adequate signal which is a desirable value (greater than 4). So the model was appropriate and fit and could be employed to navigate the design space.

**Table 3:** ANOVA for response surface quadratic model analysis of variance table.

Source	Sum of squares	Degree of freedom	Mean square	*F*-value	*p*-value
Model	6597.89	9	733.10	237.17	< 0.0001**
X_1_	677.12	1	677.12	219.06	< 0.0001**
X_2_	534.65	1	534.65	172.97	< 0.0001**
X_3_	315.01	1	315.01	101.91	< 0.0001**
X_1_X_2_	69.72	1	69.72	22.56	0.0021**
X_1_X_3_	83.72	1	83.72	27.09	0.0012**
X_2_X_3_	12.60	1	12.60	4.08	0.0832
X_1_^2^	949.58	1	949.58	307.21	< 0.0001**
X_2_^2^	2741.65	1	2741.65	886.98	< 0.0001**
X_3_^2^	752.38	1	752.38	243.41	< 0.0001**
Residual	21.64	7	3.09		
Lack of Fit	15.89	3	5.30	3.68	0.1201
Pure Error	5.75	4	1.44		
Cor Total	6619.53	16			

** Significance with *p* < 0.01

#### Response surface analysis

**Fig 3:**
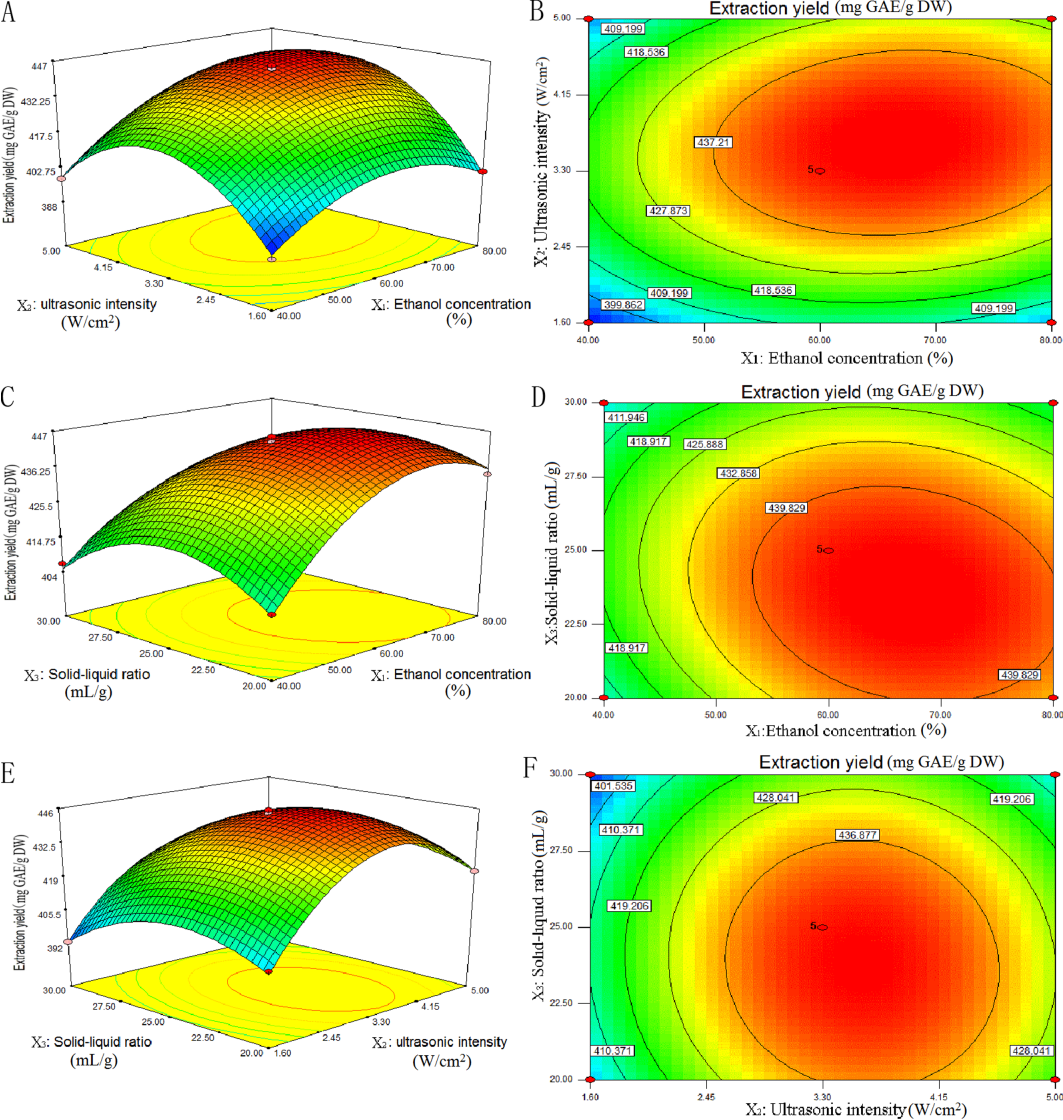
Response surface (3D) and contour plots (2D) showing the effects of different extraction parameters (X_1_: ethanol concentration, %; X_2_: ultrasonic intensity, W/cm^2^; X_3_: solid-liquid ratio, mg/mL) added on the response Y.

Two-dimensional contour plots and three-dimensional response surface displayed in Fig 3A–3F for independent variables (ethanol concentration, ultrasonic intensity and solid-liquid ratio). The results were obtained by maintaining the two variables unchanged which proved the variance in TP yield under UAE conditions.

The effects of ethanol concentration and ultrasonic intensity on the yield of TP were given in Fig 3A and 3B. The solid-liquid ratio was set at 25 mg/mL. On the whole, TP yield first increases and then decreases at different ethanol concentrations. On a smaller scale, yield of TP enhanced gently with rise in ethanol concentration at lower or higher ultrasonic intensity. However, the yield of TP markedly enhanced with rise in ethanol concentration near the central point. The change in TP yield with ultrasonic intensity also displayed a similar trend. The extraction yield of TP varied faster at higher ethanol concentration than at lower ethanol concentration. The two-dimensional contour plot (Fig 3B) was oval in shape, which indicated that the impact of interaction was significant between ethanol concentration and ultrasonic intensity on TP yield.

Fig 3C and 3D displays the effect of solid-liquid ratio and ethanol concentration on the yield of TP. The ultrasonic intensity was set at 3.3 W/cm^2^. The extraction yield of TP first increased with lower ethanol concentration, and then decreased gently with an increase in solid-liquid ratio. While, at higher ethanol concentration, the extraction yield of TP reduced quickly with a rise in solid-liquid ratio. In addition, near the central point of solid-liquid ratio, the yield of TP changed greatly with a rise in ethanol concentration. Fig 3D was also oval in shape, which proved that the interaction effect of ethanol concentration and ultrasonic intensity was significant on the yield of TP.

Fig 3E and 3F represents the influence of solid-liquid ratio and ultrasonic intensity on TP yield. The ethanol concentration was set at 60 °C. It showed that the yield of TP first enhances and then decreases at different ultrasonic intensities or solid-liquid ratios. Fig 3F was round in shape, which indicated that the effect of interaction of solid-liquid ratio and ultrasonic intensity on TP yield was insignificant.

#### Experimental validation and optimization of extraction parameters

Optimum conditions obtained by using Design-Expert 7.0.0 software for ethanol, ultrasonic intensity and solid-liquid ratio were 67.63%, 3.64 W/cm^2^ and 23, respectively. Single-factor analysis was used for the determination of other parameters. While the value of TP obtained under the above conditions was 447.8 mg GAE/g DW. While the value of TP obtained under the above conditions was 447.8 mg GAE/g DW. The conditions modified for the ease of operation are: 68% ethanol concentration, 3.6 W/cm^2^ ultrasonic intensity and a value of 23 for solid-liquid ratio. Under the above modified conditions, the value of TP obtained was 448.7 ± 2.15 mg GAE/g DW (N = 3) which was higher than the non-optimized conditions and near to the predicted value, showed that the model was fit for the extraction of TP (Table 4).

**Table 4:** Predicted and experimental values of the responses at optimum conditions.

Optimum condition			Extraction yield (mg GAE/g DW)	
Ethanol concentration (%)	Ultrasonic intensity (W/cm^2^)	Solid-liquid ratio (mg/mL)	Experimental	Predicted
68 (67.63)	3.6 (3.64)	23 (23.43)	448.7 ± 2.15	447.8

### Comparison with the soxhlet extraction method

Soxhlet extraction and UAE results of TP from dried ripe fruit of *T*. *chebula* approved that UAE gave a similar extraction yield (448.7 mg GAE/g DW) to the Soxhlet extraction value (443.5 mg GAE/g DW). However, the Soxhlet extraction approach required more solvent and consume more time than the UAE. Additionally, bioactivity of TP could be weakened by the longtime treatment under high temperature. Therefore, it could be concluded that UAE was better suited to extract TP from *T*. *chebula* fruits than Soxhlet extraction method.

### Antioxidant activity *in vitro*

**Fig 4:**
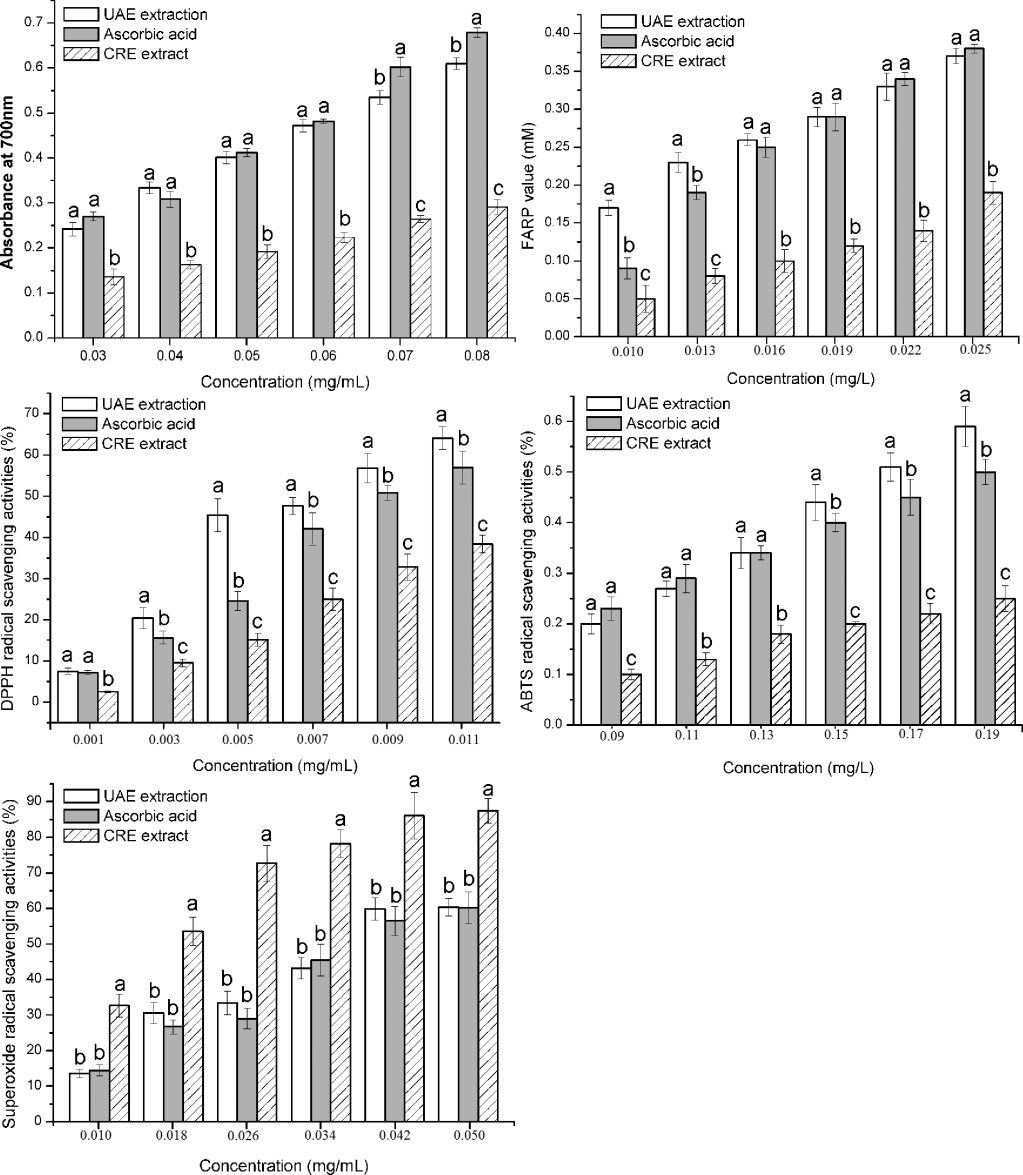
Antioxidant activities of the UAE extract, the CRE extract and Ascorbic acid. (A) Reducing power, (B) FRAP, (C) DPPH radical-scavenging activity, (D) ABTS radical-scavenging activity and (E) superoxide radical-scavenging activity. Values were expressed as mean ± SD (n = 3), and evaluated by one-way AVONA followed by the Tukey test. Different letter and same letter were considered to be statistically significant (*p* < 0.05) and statistically insignificant (*p* > 0.05), respectively.

Generally, several approaches are needed to evaluate the antioxidant activities of a plant extract. During reducing power assay, antioxidants caused the reduction of Fe^3+^ to Fe^2+^ by donating an electron. So reducing power is widely used as a potential indicator for antioxidant activity [31]. The reducing potential of Ascorbic acid, UAE extract and CRE extract are shown in Fig 4A. The reducing powers exhibited a dose-dependent response. Coefficient of determination (R^2^) for UAE extract, Ascorbic acid and CRE extract were 0.9963, 0.9831 and 0.9964, respectively. The reducing power of UAE extract and positive control were almost at the same level at 0.03–0.06 mg/mL (*p* > 0.05), while the UAE extract was lower than the positive control at 0.07–0.08 mg/mL (*p* < 0.05). As expected, the scavenging activity of the CRE extract was much weaker than UAE extract and Ascorbic acid at 0.03–0.08 mg/mL (*p* < 0.05). The slopes of the trend lines for UAE extract, Ascorbic acid and CRE extract were 7.1829, 8.5543 and 3.1714 respectively, which also indicated that the reducing power of UAE extract increased slower than Ascorbic acid and faster then CRE extract. The sample solutions with high concentration were not further tested due to the fact that absorbance should be lower than 1 for preventing unacceptable relative errors.

In order to evaluate the total antioxidant activity of *T*. *chebula* fruit extract, an accurate and simple FRAP (ferric reducing antioxidant power) assay was used [32]. As shown in Fig 4B, the FRAP of UAE extract was greater than Ascorbic acid at 0.010–0.013 mg/mL (*p* < 0.05). Nevertheless, both of them were almost at the same level at 0.013–0.025 mg/mL (*p* > 0.05). Additionally, both of them were obviously higher than CRE extract (*p* < 0.05).

DPPH free radical is a stable free radical which has been widely employed to evaluate the in vitro antioxidant activities. Fig 4C showed the scavenging effects of Ascorbic acid and *T*. *chebula* fruit extract on DPPH free radicals. The DPPH free radical scavenging activity of UAE extract was far stronger than that of Ascorbic acid at 0.003–0.011 mg/mL (*p* < 0.05). Moreover, there is a high correlation (R^2^ = 0.9130) between the DPPH scavenging activity and contents. IC_50_ value of UAE extract and Ascorbic acid was 0.0066 and 0.0088 mg/mL, respectively. These findings indicated that UAE extract had stronger DPPH radical scavenging ability than Ascorbic acid. However, the DPPH radical scavenging ability of CRE extract was weaker than Ascorbic acid at 0.001–0.011 mg/mL (*p* < 0.05).

ABTS^·+^ could generate a blue-green radical cation on oxidation. Some antioxidants can react with ABTS radical, which leads to fading of the mixture solution. Therefore, ABTS method could be used to assess antioxidant activity [33]. As shown in Fig 4D, the tested samples and Ascorbic acid showed dose-dependent activities. ABTS scavenging activities of Ascorbic acid and UAE extract were almost found at the same level at concentration ranged from 0.09–0.13 mg/mL (*p* > 0.05). However, the scavenging activity of UAE extract was stronger than Ascorbic acid at 0.15–0.19 mg/mL (*p* < 0.05). The IC_50_ obtained for UAE extract and ascorbic acid was 0.17 and 0.19 mg/mL, respectively. Intriguingly, a previous study reported the same IC_50_ value (0.19 mg/mL) for ascorbic acid [34]. These findings revealed that the UAE extract showed similar ABTS scavenging activity as Ascorbic acid. Meanwhile, it has been noted that both the UAE extract and ascorbic acid had stronger ABTS scavenging activities than the CRE extract at 0.09–0.19 mg/mL (*p* < 0.05).

The superoxide anion could be produced *in vivo* by pyrogallic acid directly under alkaline conditions. Superoxide radical can further interact with other molecules to generate hydrogen peroxide and hydroxyl radical, which caused oxidative damage in DNA, lipids and proteins [35]. Antioxidants were able to scavenge superoxide anion radicals due to its ability to reduce the reaction (auto-oxidation) of pyrogallic acid. It has been noted that the superoxide radical scavenging activity of UAE extract was similar to that of ascorbic acid at 0.01–0.05 mg/mL (*p* > 0.05) (Fig 4E). However, the result could not be explained in this paper that the superoxide radical scavenging activity of the CRE extract was stronger than that of the UAE extract or Ascorbic acid (*p* < 0.05). The IC_50_ obtained for the UAE extract, ascorbic acid and the CRE extract on ABTS radical was 0.038, 0.038 and 0.016 mg/mL, respectively. Notably, our findings showed that *T*. *chebula* fruit extract was capable of relieving the oxidative injury by the superoxide anion.

It can be seen from the above results that the antioxidant activities of the UAE extracts were stronger than that of the CRE extract except the superoxide radical scavenging activity. It has been inferred that some active compounds were destroyed by the longtime extract (Comparison with the Soxhlet extraction method), although there are similar yield between the UAE extract and the CRE extract.

### Identification of phenolic compounds

**Fig 5:**
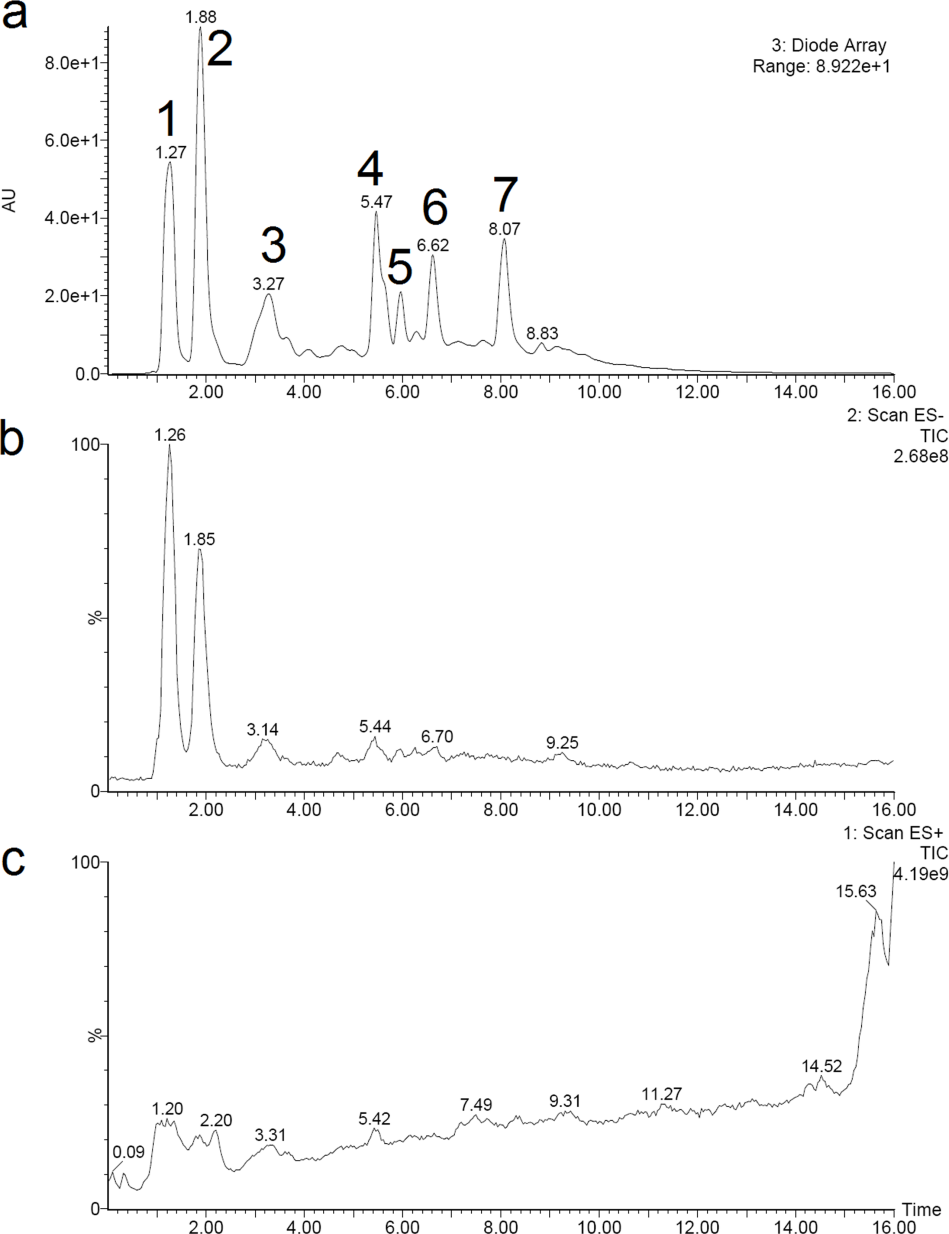
**The HPLC chromatogram (a), total ion chromatogram of mass spectrometer in negative ion mode (b) and positive ion mode (c) of the T. chebula fruits extract**.

**Fig 6:**
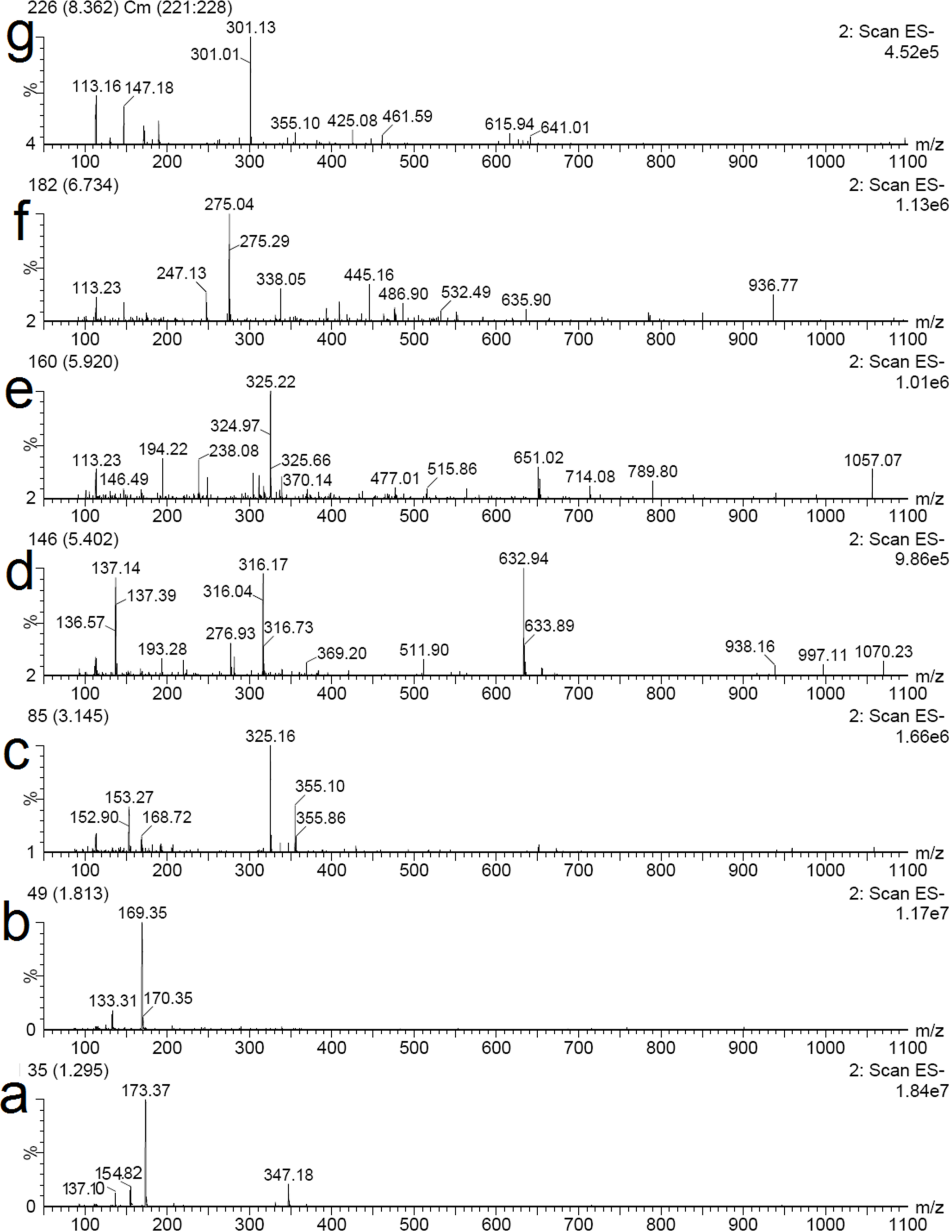
**The MS spectra in negative mode of seven representative compounds in the *T*. *chebula* fruits extract**: shikimic acid (a), gallic acid (b), 5-O-galloylshikimic acid (c), corilagin (d), peak (e), 3,4,8,9,10-Pentahydroxydibenzo [b, d] pyran-6-one (f), ellagic acid (g).

**Fig 7:**
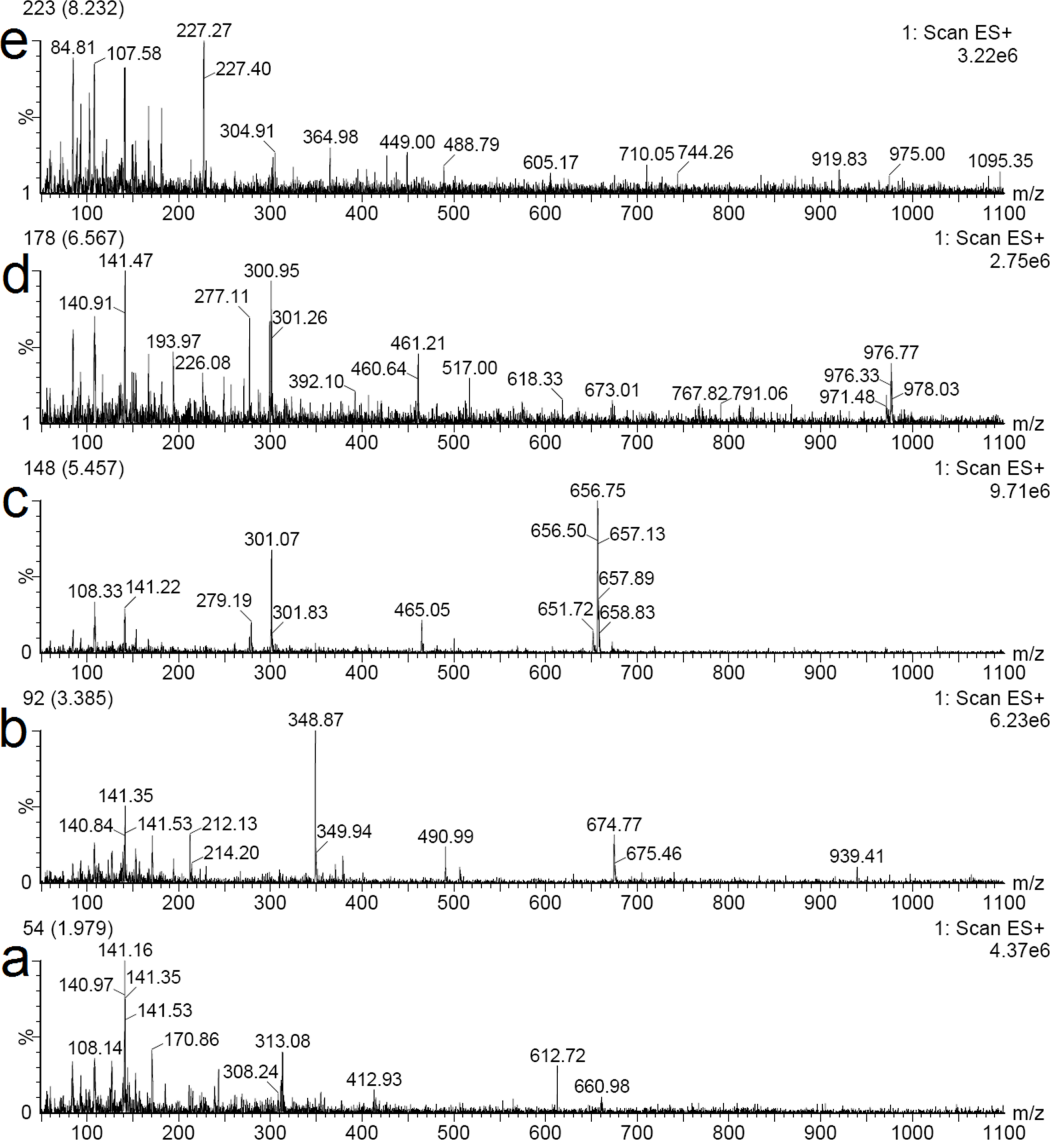
**The MS spectra in postitive mode of seven representative compounds in the *T*. *chebula* fruits extract**: gallic acid (a), 5-O-galloylshikimic acid (b), corilagin (c), 3,4,8,9,10-Pentahydroxydibenzo [b, d] pyran-6-one (d), ellagic acid (e).

In order to identify the structures of main constituents in the *T*. *chebula* fruits extract, the sample was analyzed by HPLC–ESI-MS techniques in both negative and positive mode. Fig 5 showed that phenolic acids were detected sensitively in negative mode. The detected constituents exhibited their quasi-molecular ions [M–H]^−^. By careful studying on the mass spectra of these compounds (Figs 6 and 7) and comparing with reference data, 6 peaks in the *T*. *chebula* fruits extract were designated and identified (Table 5). They were shikimic acid (peak 1), gallic acid (peak 2), 5-O-galloylshikimic acid (peak 3), corilagin (peak 4), 3,4,8,9,10-Pentahydroxydibenzo [b, d] pyran-6-one (peak 6), ellagic acid (peak 7), respectively.

**Table 5:** HPLC-ESI-MS fragmentation (negative and positive ion mode) of the compounds detected in *T*. *chebula* fruit extract.

	R_t_ (min)	Negative ion mode	Positive ion mode	Mol. weight
Peak 1	1.27	173.37 [M-H]^-^		174
		154.82 [M-H_2_O-H]^-^		
		137.10 [M-H_2_O-H_2_O-H]^-^		
Peak 2	1.88	169.35[M-H]^-^	170.86 [M+H]^+^	170
		781.02[M-H]^-^	141.50 [M+H-CO]^+^	
Peak 3	3.27	325.16 [M-H]^-^	348.87 [M+Na]^+^	326
			674.77 [2M+ Na]^+^	
Peak 4	5.47	632.94 [M-H]^-^	656.75 [M+ Na]^+^	634
		316.17 [M-2H]^2-^		
Peak 5	5.92	325.22 [M-H]^-^		326
		651.2 [2M-H]^-^		
Peak 6	6.62	275.04 [M-H]^-^	277.11 [M+H]^+^	276
		247.13 [M-H-CO]^-^		
Peak 7	8.07	301.13[M-H]^-^	605.17[2M+ H]^+^	302

Note: Peaks 1–7 represented shikimic acid, gallic acid, 5-O-galloylshikimic acid, corilagin, Peak 5 (unknown), 3,4,8,9,10-Pentahydroxydibenzo [b, d] pyran-6-one and ellagic acid, respectively.

The mass spectrum of Fig 6A showed the negative quasi-molecular ion at m/z 173.37 [M-H]^-^ and product ions at m/z 154.82 [M-H_2_O-H]^-^, 137.10 [M-H_2_O-H_2_O-H]^-^, which is similar to the characteristics of shikimic acid [36]. The mass spectrum of Fig 6B and Fig 7A showed the negative quasi-molecular ion at m/z 169.35 [M-H]^-^ and the positive quasi-molecular ion at m/z 170.86 [M+H]^+^ and the signal at m/z 141.50 [M+H-CO]^+^ due to the loss of CO, which corresponds to the fragmentation regularity of gallic acid [37]. The mass spectrum of 5-O-galloylshikimic acid confirmed the presence of the following signals: the negative quasi-molecular ion [M-H]^-^ at 325.16 m/z (Fig 6C), and the positive quasi-molecular ion [M+ Na]^+^ at 348.87 m/z and [2M+ Na]^+^ at 674.77 m/z (Fig 7B) [38]. The mass spectrum of corilagin confirmed the presence of the following signals: the negative quasi-molecular ion at [M-H]^-^ at 632.94 m/z (Fig 6D) and [M-2H]^2-^ at 316.17 and the positive quasi-molecular ion [M+ Na]^+^ at 656.75 m/z (Fig 7C). The ESI-MS spectrum of Fig 6E in addition to the quasi-molecular ion [M-H]^-^ at m/z 325.22, shows another fraction ion at m/z 651.2 [2M-H]^-^, the molecular mass of the compound is 326. The ESI-MS spectrum of Fig 6F represented the quasi-molecular ion [M-H]^-^ at m/z 275.04, and Fig 7D showed the quasi-molecular ion [M+ Na]^+^ at 277.11 m/z, which corresponds to the characteristics of 3,4,8,9,10-Pentahydroxydibenzo [b, d] pyran-6-one [39]. Finally, the mass spectrum of ellagic acid showed the negative quasi-molecular ion [M-H]^-^ at 301.13 m/z (Fig 6G) and the positive quasi-molecular ion [2M+H]^+^ at 605.17 (Fig 7E) [40].

Manna, K., et al reported that shikimic acid reversed the H_2_O_2_ induced oxidative damage in hepatocytes by inhibition of NF-kappa B, activation of PI3K/Akt/Nrf2 pathway and reduction of apoptosis [41]. Moreover, it has been proved that gallic acid, 5-O-galloylshikimic acid, corilagin, 3,4,8,9,10-Pentahydroxydibenzo [b, d] pyran-6-one and ellagic acid had significant antioxidant activities [42–44]. Therefore, these compounds could be responsible for the antioxidant activities of *T*. *chebula* fruits extract.

## Conclusions

RSM was successfully applied to optimize the extraction parameters for total phenolics (TP) yield from the *T*. *chebula* fruit. The optimal combination was determined to be an ethanol concentration of 68%, ultrasonic intensity of 3.6 W/cm^2^, solid-liquid ratio of 23 mg/mL, particle size of 0.18 mm and ultrasonic time of 20 min for 2 times at 70 °C. The yield of TP reached 448.7 ± 2.15 mg GAE/g DW under these conditions. Besides, the antioxidant activities of the UAE extract were stronger than that of the CRE.
